# The longest path in the Price model

**DOI:** 10.1038/s41598-020-67421-8

**Published:** 2020-06-29

**Authors:** Tim S. Evans, Lucille Calmon, Vaiva Vasiliauskaite

**Affiliations:** Centre for Complexity Science and Theoretical Physics Group, Physics Department, Imperial College London, London, SW7 2AZ UK

**Keywords:** Complex networks, Applied mathematics

## Abstract

The Price model, the directed version of the Barabási–Albert model, produces a growing directed acyclic graph. We look at variants of the model in which directed edges are added to the new vertex in one of two ways: using cumulative advantage (preferential attachment) choosing vertices in proportion to their degree, or with random attachment in which vertices are chosen uniformly at random. In such networks, the longest path is well defined and in some cases is known to be a better approximation to geodesics than the shortest path. We define a reverse greedy path and show both analytically and numerically that this scales with the logarithm of the size of the network with a coefficient given by the number of edges added using random attachment. This is a lower bound on the length of the longest path to any given vertex and we show numerically that the longest path also scales with the logarithm of the size of the network but with a larger coefficient that has some weak dependence on the parameters of the model.

## Introduction

The Price model^1,2^ is one of the oldest network models and it was motivated by the pattern of citations in academic papers. In a citation network, each node represents a document while every entry in the bibliography of a document *t* is represented by a directed edge from an older document, node *s*, to node *t*. One of the key features of a citation network, one inherent in the Price model, is that there is a fundamental *arrow of time* in the network; bibliographies can only refer to older documents. This means that there are no cycles in the network, you can never find a path from a node that returns to that node. Thus a citation network is an example of a Directed Acyclic Graph (DAG).

**Fig. 1:**
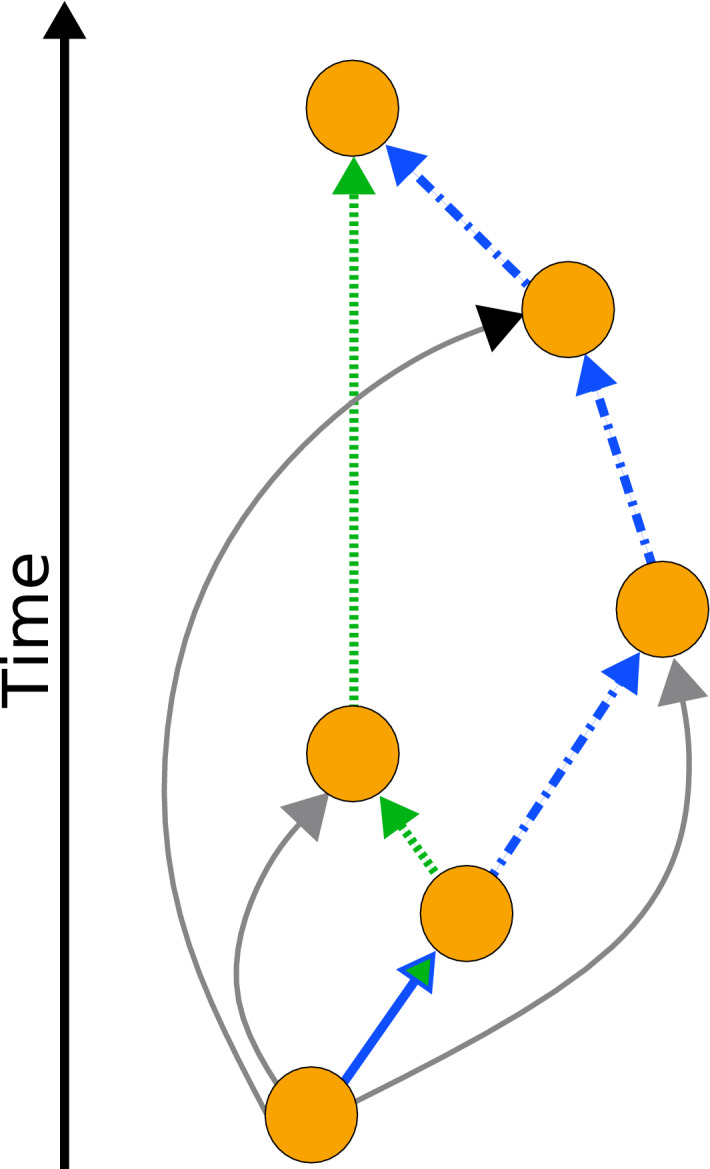
An illustration of a Price-model style DAG where the longest, shortest and reverse greedy paths from last point to the first are distinct. The longest path from the source node to the sink node is highlighted in blue dot-dash line; the reverse greedy path is the dotted green path. Note the first edge is the same for both—the green-blue edge. As illustrated here, the Price model produces DAGs which are neither transitively complete nor transitively reduced. In a transitively complete DAG, all nodes which are connected by a path are connected by a direct edge. Likewise, except for the case of one-incoming edge per node, the model is not transitively reduced^3^, that is some edges could be removed without removing a path between any pair of nodes.

Mathematically, DAGs have some distinctive properties and one of them is that for any pair of connected nodes there is a well defined and meaningful longest path length, for example see Fig. 1. Contrast this with, for example, undirected networks, where you can often find many paths between two given vertices that visit most of the nodes in a component so longest paths are often as long as the component is big, if all nodes in the path must be distinct, and infinite, if multiple visits to the same node were allowed. In directed graphs with cycles, the longest path is infinite, if multiple visits to a node are allowed. Both of these definitions of the longest path coincide if the network is acyclic, as the absence of cycles ensures that in any path, a node can only occur once.

In a citation network, it is not clear how useful the shortest path is. For instance, in writing this paper, the oldest citation we have is to a paper by Price^1^. The shortest path to Price’s paper from this work has length one. On the other hand, most of the knowledge of that work contained in this paper did not come directly from that paper. We only reread Price’s paper to check one detail while working on this project. So the length of the shortest path to that paper seems largely irrelevant. Rather, the information in this early bibliometrics paper by Price has reached us through a sequence of other work, much of it not explicitly referenced in our paper. We drew on much more recent documents such as the reference book by Newman^4^ which in turn cites papers which developed various aspects of the Price model. Indeed there is much evidence^3,5,6^ that typically 70% or so of a bibliography may not have been used directly when producing the work in an academic paper.

So our thesis is that for DAGs the longest path plays a much more important role than the shortest path. In simple models the longest path has been shown to be the best approximation to the geodesic for models of DAGs embedded in Minkowski space^7^ where there is a single time direction. This has been exploited in real data sets where dimension and curvature of a DAG can be measured^8,9^ enabling us to embed DAGs such as citation networks in Minkowski space^10^. A similar rigorous link for undirected networks has only been made for the shortest path in networks embedded in Euclidean space where there is no arrow of time^11,12^].

The properties of the longest path have been investigated in the context of simple models known as Cube Spaces^13^ which include those built from Poisson Point Processes in Minkowski spaces where all causally connected points are connected to form a network. However these are examples of transitively complete DAGs, that is if there is a path between two points then there is always an edge connecting those two points directly. However, that is not true for a citation network where the limited size of a bibliography means no document ever cites every older paper to which it has some connection. What we seek to do in this paper is to look at the properties of the longest path in a simple model, the Price model^2^ and its variations, where the network is neither transitively complete nor (except for one parameter value) transitively reduced, the situation for most DAGs in a social context such as citation networks. Can we calculate the length of the longest path in the Price model? How does this length depend on the parameters of the model?

We start by outlining our analytic results. We then compare these predictions to numerical simulations and summarise our findings.

## Analytic results

**Fig. 2:**
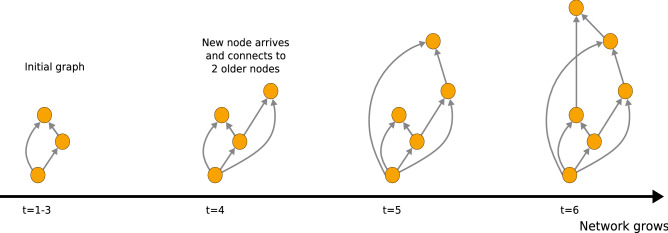
An illustration of the Price Model. Here the height of the node on the page indicates the time with the first node at the bottom being the node $t=1$. At each stage we show the graph $\mathcal {G}(t)$ so after the new nodes and its incoming edges have been added.

In the Price model^2^ (for instance see Sect. 14.1 of Newman^4^) we start from a network *G*(*t*) defined at an integer ‘time’ *t*. We create a new graph $G(t+1)$ by first adding one new vertex, which we label with the time $(t+1)$. This new node, $(t+1)$, is connected to *m* existing vertices *s* in the graph *G*(*t*). These *m* existing vertices $\{s\}$ are each chosen with probability $\Pi (t,s)$. We will use a convention that these edges point from older to newer vertices, from *s* to $(t+1)$. Once these edges have been added we have our new graph $G(t+1)$. The process is then repeated. For an example of how a network grows according to the Price model, see Fig. 2.

The mathematical and numerical simplicity of this model comes from the simple definition of $\Pi (t,s)$. To define the probability $\Pi (t,s)$ we first define $N(t)=N_0 + t$ be the number of nodes in the graph *G*(*t*) for some constant $N_0$. The number of edges in the graph *G*(*t*), after all *m* edges have been added to node *t*, is $E(t)=E_0 + mt$ where $E_0$ is some constant. Finally in the graph *G*(*t*) let the node created at time *s* have out-degree $k^{(\mathrm {out})}(t,s)$, the number of edges leaving *s* and connecting it to later nodes.

In this model, the connection of edges to new node $(t+1)$ is made in one of two ways. With probability *p* node $(t+1)$ is connected to an existing vertex *s* chosen in proportion to the number of edges leaving *s* for later nodes in *G*(*t*), $k^{(\mathrm {out})}(t,s)$. Price called this cumulative advantage and, after normalisation, we have that the probability of choosing *s* is $k^{(\mathrm {out})}(t,s)/E(t)$. The second process happens with probability $\bar{p}=(1-p)$ and in this case we choose the source vertex *s* uniformly at random from the set of vertices in *G*(*t*), i.e. with probability 1/*N*(*t*). If we start the process at time equal to 1, then the probability of connecting the vertex $(t+1)$ to existing vertex *s* is $\Pi (t,s)$ where

1 $$\begin{aligned} \Pi (t,s) = p \frac{k^{(\mathrm {out})}(t,s)}{E(t)} + \bar{p}\frac{1}{N(t)} \text{ if } t \ge s \ge 1 \, , \end{aligned}$$

unless $s=t=1$ when $\Pi (t,s)=1$, otherwise $\Pi (t,s)=0$. Note that in his original paper, Price considered $p=m/(m+1)$ where $\Pi \propto k^{(\mathrm {out})}+ 1$. This more general form for the attachment probability $\Pi (s,t)$ in Eq. (1) has been used in many related contexts since Price, see Newman^4^ for a review.

There is an issue about the starting point for this process. The usual form for $\Pi$, the $t\ge s > 1$ form in Eq. (1), leaves us with a problem for $\Pi (t=1,s=1)$ when looking at the attachment to the second vertex, $t=2$. Our solution is to demand that $\Pi (t=1,s=1)=1$. This fixes the cumulative probability $\Pi _\le$ to have a consistent value which is in fact all we need for this calculation. However, we will also assume that the number of nodes and number of edges are given by $N(t)=t$ and $E(t)=mt$ respectively. This is only needed for $t\ge 2$ so in principle we must allow multiple edges between nodes starting with *m* edges added between node 2 and node 1. Again this cannot be true for at least the first node at $t=1$.

We also note that our analytic calculations allow our networks to contain multiple edges (node pairs linked by more than one edge). Of course, a real citation network and many numerical calculations of this model (though not our numerical calculations) do not have multiple edges. However, in the long time limit the effect of such edges becomes negligible as they form a small fraction of the edge population, a fraction that dies off as a power law in time^4^.

Now we would like to define the longest path algebraically. Unfortunately, finding the longest path requires global knowledge of all the paths. This is extremely hard to do algebraically (though is surprisingly straightforward numerically). So the first stage of our calculation is to decide to calculate a path defined with local knowledge only. That is we define what is called a reverse greedy path using an iterative process where at each stage we only need to know about the properties of the next vertex in the path. We will denote the length of the reverse greedy path from the source vertex $s=1$ to a target vertex *t* as $\ell (t)$. The length of the longest path from the source vertex to a target vertex *t* will be denoted as *L*(*t*).

The reverse greedy path to a node *t* is a path running from the source node at the initial time $t=1$ to node *t*. This always exists and it is unique. To define it suppose that we have found the reverse greedy path to all earlier nodes. The last step on the reverse greedy path to node *t* is made along the edge arriving at *t* from its most recent predecessor node, say *s*. The idea is that the most recent predecessor of node *t*, furthest from the source node in terms of the time, is also the most likely to be the predecessor node furthest from the sink node in terms of network path lengths. There is no guarantee that our reverse greedy path is identical to the longest path, so the reverse greedy path length is a lower bound on the longest path length. A more formal definition is given in Appendix A.1 in the Supplementary Information.

Of course in any one instance of the Price model, this reverse greedy path length will fluctuate if we look at nodes of similar ages, not least because $\ell$ is integer valued. We will use a mean field approach so our $\ell (t)$ is an average over many realisations of the model though for simplicity we will not include the expectation value notation $\langle \ldots \rangle$. For that reason our $\ell (t)$ will be a real valued monotonically increasing function of time *t*.

We can find the long-time behaviour using the following simple argument. On average, there are *pm* edges added with cumulative advantage at each time step. Suppose we are adding a new node at time $(t+1)$ and we are looking for source nodes *s* for the *m* edges we are going to add. The source nodes chosen with cumulative advantage are those with largest degree and those are the oldest nodes created at small values of time *s* (since $k^{(\mathrm {out})}(t,s) \propto (t-s)^p$, for instance see Newman^4^). So the youngest source node chosen, nodes created at the largest value of time *s*, is likely to be one of the $m_{{{\text{rnd}}}} = m\bar{p}$ nodes chosen uniformly at random. The probability that all these $m_{{{\text{rnd}}}}$ randomly chosen source nodes are chosen between time 1 and time $\hat{s}$ inclusive is $(\hat{s}/t)^{{m_{{{\text{rnd}}}}}}$. Suppose we consider the time $\hat{s}_{1/2}$ where with probability one half the time coordinate of the largest randomly chosen source node is $\hat{s}_{1/2}$ or less, then this sets the scale for the birth date of the youngest source node connected to node $(t=1)$, namely that $\hat{s}_{1/2} = \mu t$ where $\mu = 2^{{ - m_{{{\text{rnd}}}}}}$. This is the previous node on the reverse greedy path from the initial node to node *t*. We can then estimate the numbers of steps it takes to get back to the source node at $t=1$ as $\mu ^{\ell } t \approx 1$ which leads to

2 $$\begin{aligned} \ell (t) = \frac{m \bar{p}}{\ln (2)} \ln ( t ) \, . \end{aligned}$$

The simplicity of the attachment probability in the Price model means we can also produce more detailed derivation using a mean-field approach. Let the probability that the length of the reverse greedy path, $\ell$, from new node $(t+1)$ to the initial node at $t=1$, be $P(\ell ,t)$. Then the master equation is of the form

3 $$\begin{aligned} P(\ell ,t+1)= & {} \sum _{s=1}^{t} P(\ell -1,s) \Pi _{\mathrm {max}}(t,s) \, . \end{aligned}$$

Here $\Pi _{\mathrm {max}}(t,s)$ is the probability that of the *m* predecessor nodes connected to a new node at $(t+1)$, the oldest of them is *s*. In terms of the generating function $G(z,t) = \sum _{\ell =0}^\infty z^\ell P(\ell ,t)$ we find that the exact solution in the Price model is (see Appendix A.3 in the Supplementary Information for details)

4 $$\begin{aligned} G(z,t)= & {} \prod _{s=1}^{t-1} \left( z \left( 1 - \left( \frac{s-\bar{p}}{s} \right) ^m\right) + \left( \frac{(s-\bar{p})}{s} \right) ^m \right) . \end{aligned}$$

Exact forms for the expected reverse greedy path length can be found from this expression, especially for specific small values of *m*. However, the leading order contribution for large times is always of the form

5 $$\begin{aligned} \lim _{t \rightarrow \infty } \ell (t)= & {} m\bar{p}\ln (t) - m\bar{p}\psi (m\bar{p}+ 1) + \sum _{n=2}^{m} \left( {\begin{array}{c}m\\ n\end{array}}\right) (-1)^{n-1} (\bar{p})^n \zeta (n) + O(t^{-1}) \end{aligned}$$

where it is implicit that there is no contribution from the term with the sum for the case of $m=1$. Here $\psi (z)$ is the digamma function and $\zeta (z)$ is the Riemann zeta-function. The details of the calculation are given in Appendix A.3 of the Supplementary Information.

Finally, the scaling properties of the longest path in the Price model suggests that the properties of height antichains are also very simple. The height of a node in a DAG is the length of the longest path to a node from a source node, any node with zero in-degree. Thus in the Price model, the height of a node is simply the length of the longest path length from the initial node to the given node, our $L$.

Nodes connected by a path cannot be of the same height. Thus the subset of all nodes at the same height form an antichain, a set of nodes in which no two are connected by a path^14^. The scaling properties of these height antichains are simple to estimate if we conjecture that the average longest path $L$ of a node *t*, its average height, scales as $\ln (t)$. This suggests that if the median index of a node in an antichain of integer valued height *h* is $t_{{{\text{mid}}}} = (\mu )^{h}$ then the mean index of nodes in the antichain will scale as $\cosh (\sqrt{\mu })(\mu )^h$, the variance in the index of nodes in the antichain will be roughly $(1/\sqrt{3}) \sinh (\sqrt{\mu }) (\mu )^h$, and the number of nodes in the antichain will vary as in the $2\sinh (\sqrt{\mu }) (\mu )^h$.

## Numerical methods and results

In the master Eq. (3), multiedges (attaching two edges from the new vertex to the the same vertex) are not excluded. In our numerical implementation code we also allowed multiedges to be created. However the probability of attaching one edge from new vertex $(t+1)$ to any existing vertex *s* is decreasing as $(s/t)^{p}$ (for instance see p. 489–90 Newman^4^). So the creation of a multiedge becomes negligible at large times hence our networks are essentially the same as implementations of the Price model in which multiedges are excluded.

The first few steps of the numerical implementation of the Price model have some subtleties which are worth mentioning. The problems noted analytically with the initial node at $t=1$, which is the only node with zero-in-degree, exemplify the issue. The earliest nodes are those with the shortest values of our $\ell$ and $L$ path lengths to the first node. Since the path lengths of the first few nodes will be added to any other path routed via one of these early nodes, we expect the initial graph to give a constant contribution to the path lengths we measure but not to alter the growth in length scales over long-times.

We chose to start our simulations from a complete graph of $(2m+1)$ nodes, labelled $t=1$ to $t=(2m+1)$. All pairs of nodes are connected in this initial graph, with the edge direction from earlier to later node. This initial graph ensures that $E(t)=mt$ for all graphs generated numerically, *G*(*t*) for $t\ge (2m+1)$. The out-degree distribution is fairly even but the in-degree is not fixed to be *m* for nodes in this initial graph. Further notes on the effect of the initial graph are given in Appendix B.4 in the Supplementary Information.

In order to simplify and accelerate the numerical analysis, for each new node $(t+1)$ added we drew nodes uniformly at random from an “attachment list” which we maintain. After we have chosen the source edges for the *m* edges attached to the new vertex, we update our attachment list by adding each source node once for every edge, and we add $(\bar{p}/pm)$ references to the new node. This means we restrict our results to cases where $(\bar{p}/pm)$ is an integer. Drawing nodes uniformly from our attachment list means that we are choosing vertices according the the probability Eq. (1). For the special case where there was no cumulative advantage, $p=0$, the attachment list was simply a list of the existing vertices where each is referenced once. A more detailed explanation is given in Appendix B.1 of the Supplementary Information.

For each node *s*, we also record values for the lengths of the reverse greedy path $\ell (s)$ and the longest path $L(s)$. When adding a new node *t*, it is simple to look at the values of the lengths of these paths to the *m* nodes attached to the new node. From that information, it is simple to record the lengths of these paths to the new vertex, $\ell (t)$ and $L(t)$. Storing and manipulating these results proved to be more of a limiting factor than the speed to produce them. We produced results for networks of up to $10^8$ nodes.

**Fig. 3:**
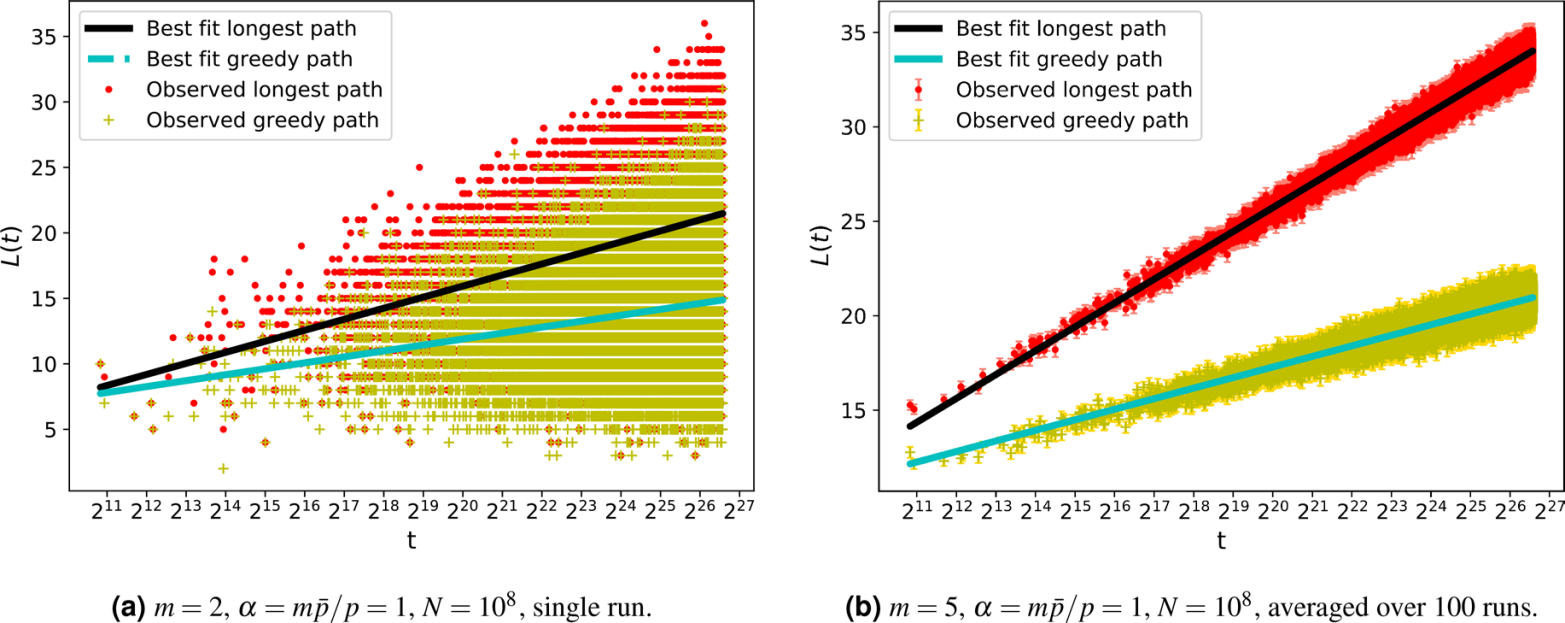
On the left is a plot of path length against *t* for a single network realisation showing noisy the data is. Averaging over 100 runs greatly reduces the fluctuations as shown in the righthand plot. Fitted lines are of the form $a \ln (t) +b$. In both figures, a random sample of $10^5$ points is plotted. The abscissa axes are logarithmic to show the linear behaviour between the path lengths and *t*.

The results for these path lengths are quite noisy for any one node as shown by an exemplary run in Fig. 3. Despite the relatively large fluctuations in results for any one node, there is a clear trend in the nodes created at later times. The fluctuations of the path length scaling are greatly reduced when averaged over multiple networks as shown in Fig. 3. So we use 100 runs for each set of parameter values in our work.

**Fig. 4:**
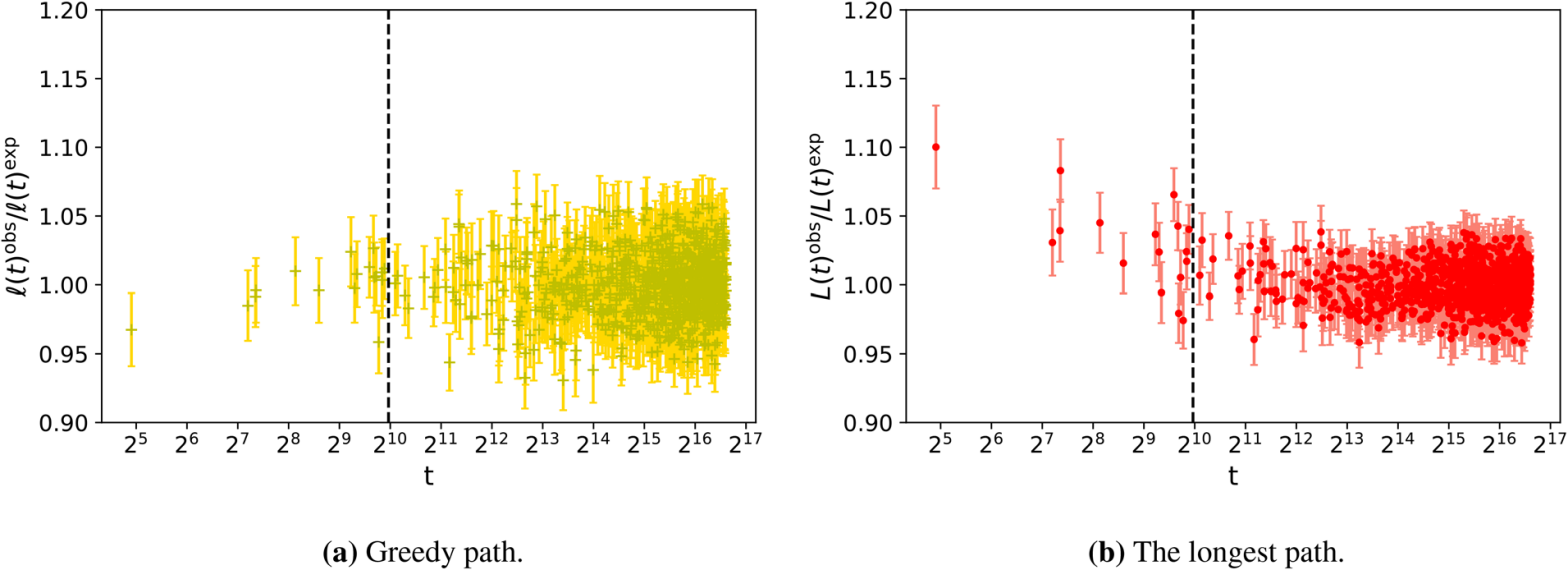
The ratio of the average over 100 runs of the path length measured numerically, ${L}^{\mathrm {obs}}$ and ${\ell }^{\mathrm {obs}}$, divided by the expected values, as described by the numerical best fit. The data for $p=0.375 \, m=5$ from $t=1,000$ (represented by a vertical dashed line) to $t=10^8$ was fitted to Eq. (6). The reverse greedy path results are shown on the left, and the longest path are shown on the right. The error bar on each point is calculated from the standard error in the mean from the results for each node over 100 runs. A random sample of $10^3$ points is plotted in both figures.

In order to compare our numerical data with the analytical results we fitted the path lengths found to the function *f*(*t*) where

6 $$\begin{aligned} f(t) = a\ln (t) + b \, . \end{aligned}$$

The fit was made by using a non-linear fitting routine based on the optimisation of the chi-squared measure of goodness of fit (for instance see^15^) as described in more detail in Appendix B.3 of the Supplementary Information. Errors on parameters were estimated from the covariance matrix produced by such a method. Given that our analytical work only studies the long-time limit and that the early times in the numerical simulation do not satisfy all the conditions of the analytical work, it is not surprising that in Fig. 4 we still see significant difference between the numerical results and analytical predictions for the length of paths from $t=1$ to those nodes created at early times. So when fitting to our numerical data we only use data for nodes created from time $t_0=1,000$ up to the last node at $t=10^8$. The effect of this cutoff is discussed in Appendix B.3 but we found varying this lower cutoff had little effect on our results since we had so many data points from the region where the asymptotic growth dominates.

**Fig. 5:**
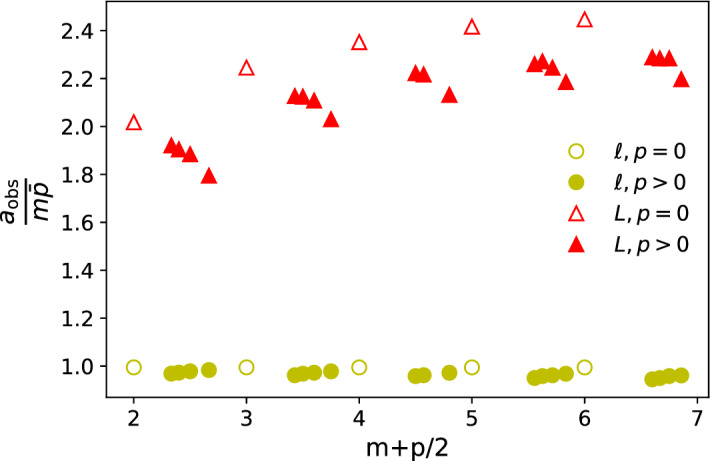
The ratio of $a_{\mathrm {obs}}/(m\bar{p})$ where $a_{\mathrm {obs}}$ is the coefficient of $\ln (t)$ derived from the best fit of the numerical path length data to $a\ln (t) + b$ Eq. (6) while $m\bar{p}$ is the analytical prediction for the value of *a* when looking at the length of the reverse greedy path. The red triangles show the results for the reverse greedy path value of *a* while yellow circles are the longest path values. These values were obtained by fitting the form to nodes created between $t=1,000$ and $t=10^8$ from 100 realisations. Errors on the fitted values of *a* were smaller than the marker size and are not shown.

**Fig. 6:**
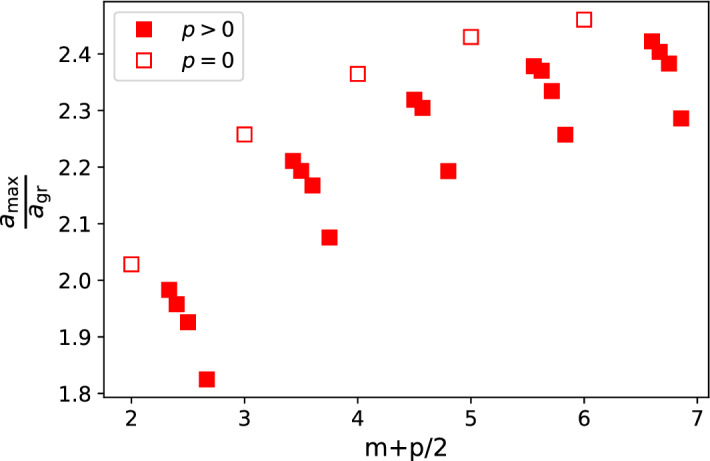
The ratio of $a_{\mathrm {max}}/a_{\mathrm {gr}}$ where *a* is the coefficient of $\ln (t)$ in the best fit of the numerical path length data to Eq. (6), $a_{\mathrm {max}}$ for the longest path data and ${a_{\mathrm {gr}}}$ for the reverse greedy path data. These values were obtained by fitting the form to nodes created between $t=1,000$ and $t=10^8$ from 100 realisations. As a result the errors on the fitted values of *a*, as estimated from the covariance matrix of the linear fitting algorithm, were smaller than the marker size and so these are not shown.

The dependence of the coefficient of the $\ln (t)$ term found from the fit, *a*, on the model parameters is shown in Figs. 5 and 6.

The next-to-leading order coefficient, *b* of Eq. (6), showed no clear trends. We also considered a non-linear fit with a term of *c*/*t* added to the expression in the Eq. (6). We found that in practice, this term had little influence on the remaining parameters of interest, namely, *a* and *b*. Furthermore, the errors in *c* were found to be relatively large in comparison to the errors of the parameters *a*.

Finally, it is clear that the longest path length is scaling as $\ln (t)$ to a good approximation. As noted above this then implies that the properties of height antichains in the Price model should follow a regular pattern which depends on the height of nodes in each antichain. Numerical confirmation of these patterns are given in Appendix B.6 of the Supplementary Information.

## Discussion

The numerical results for the leading behaviour of the path length scales are striking. Within the margin of numerical error, our results in Fig. 5 show that the length of the reverse greedy path scales asymptotically as $m\bar{p}\ln (t)$ for a wide range of parameter values. This is consistent with both the simple argument and the detailed analytical calculation presented in the Analytic Results (see also Appendix A of the Supplementary Information). The analytical approach also shows that for long times, the distribution of lengths of reverse greedy paths in the Price model is Poisson distributed with mean equal to $m\bar{p}\ln (t)$ (see Appendix A).

The reverse greedy path length is a lower bound on the length of the longest path so it is no surprise that the longest path length also scales as $\ln (t)$ with a coefficient, $a_{\mathrm {max}}$, which is larger than the corresponding scaling factor for the reverse greedy path length, $a_{\mathrm {gr}}$. Interestingly this coefficient of the $\ln (t)$ term, $a_{\mathrm {max}}$, for the longest path shows some additional weak dependence on the parameters beyond the $m\bar{p}$ found for the reverse greedy path, as both Figs. 5 and 6 clearly show.

The Price model is not in itself a very realistic model for any particular context. For instance, a true citation network often shows many other features such as a preference to cite recent papers, for example see^6,16–21^. The choice of a simple linear form for the attachment probability Eq. (1) appears to be part of this simplification, a form linear in degree motivated by the need for mathematical simplicity. At first sight, this form seems unrealistic since it requires authors of papers to have global information about the citation network because of the normalisation factors. No author can know exactly how many citations a paper has let alone the total number of citations in the network. However, this form emerges naturally in many situations as the result of doing local searches on the network, see^6,21–27^ and references therein. In more realistic models, the cumulative advantage, the *p* term in $\Pi$ emerges from doing a local search back through the current citation network, while the random attachment, the $\bar{p}$ term in $\Pi$, represents a simple model of other possible processes. So like all good models, the emphasis in the Price model on the linear form for $\Pi$ in terms of degree does capture an important and realistic feature of many real situations. This linear form of the attachment probability $\Pi$ is also the critical feature in the analysis of undirected versions of this model, such as the Barabási–Albert model^28^ where the cumulative advantage aspect is known as preferential attachment and the original example worked with $p=1/2$ in our notation.

However, the Price model, simple as it is, also emphasises another critical aspect of a citation network, and that is the inherent arrow of time in this context. Citations (almost) always point backwards in time. Typical data sets^3,8^ suggest that less than 1% of citations are to documents which are labelled as being published later than the citing document. The networks created in the Price model are realistic in this way, they always produce directed acyclic graphs. This acyclic property is lost when the edge direction is ignored, as in the Barabási–Albert implementations of this model. Since many analyses work in the undirected version, they have missed this key feature of the Price model and of real-world citation networks.

For instance, the length of the shortest path between two nodes is a natural measure for undirected networks since in some circumstances it can be related to the geodesic of networks embedded in Euclidean space, for example see^11,12^. For an undirected version of the Price model, the LCD model of Bollobás and Riordan^29,30^ (a more precisely defined version of the Barabási–Albert model^28^), it is known that the diameter, the largest length of any shortest path between two nodes, scales as $\ln (t)/(\ln \ln (t))$ if $m>1$ while the diameter scales as $\ln (t)$ for the special case of $m=1$^29^ (see also theorem 18 of^30^). For the case $m>1$, Bollobás points out that while in any random graph we expect to see the small-world effect^31^ and a $\ln (N)$ scaling of lengths (for *N* nodes in the network), for the undirected version of this model “one might expect the diameter to be even smaller: the unbalanced degree distribution pushes up the number of small paths, and thus, perhaps, pushes the diameter down” (see Bollobás^30^ section 13, p 25). That is, the unusual slow scaling of the shortest path distance scale in this undirected version of this model is due to the effect of the very high degree nodes created because of the cumulative advantage (preferential attachment) process.

However, the situation is completely different when we take account of the direction of edges in this model. First, the link between shortest path lengths and geodesics in Euclidean space used in Ref.^11,12^ is lost. The natural order of nodes in a DAG, the arrow of time, means we should compare the path lengths network against geodesic lengths for network models embedded in Minkowski space, and indeed there is a proven relationship between these two^7,10,32^. Following on from this, when using the longest path in the directed form of these models, our analysis has shown that the longest path is likely to be created by edges created from random attachment not those formed using the cumulative advantage mechanism, the opposite of what is suggested for the shortest path in the undirected form of these models. Thus the fat-tailed nature of the degree distribution in the Price model (or directed versions of the Barabási–Albert/LCD models) is not a factor for the longest path and so, using Bollobás’ insight^30^, we should expect the longest path to scale simply as $\ln (t)$, and not something slower than that. That is, indeed what we have shown in our work here.

Looking more widely, we note that Bollobás^30^ (p 10) suggested that “For these models the orientation is not very interesting”. Our conclusion is the opposite. Namely that for any directed network in which vertices are added sequentially, the arrow-of-time inherent in these growing network models is both physically relevant and this vertex order produces new and distinctive features. Our analytical and numerical analysis of the longest path length is just one illustration of what is possible.

## Supplementary information

Supplementary information.
