# Supplementary material for: The longest path in the Price model
Source: Sci Rep. 2020 Jun 29;10:10503. doi: 10.1038/s41598-020-67421-8 (PMC7324613; doi:10.1038/s41598-020-67421-8)
Supplement: Supplementary file 1 — Supplementary information. [file 41598_2020_67421_MOESM1_ESM.pdf]

Supplementary Information for Longest Path in the Price Model

T.S. Evans, L. Calmon, V. Vasiliauskaite
Centre for Complexity Science, and Theoretical Physics Group,
Imperial College London, SW7 2AZ, U.K.

15th January 2020

A Full Analytic Calculation

In this appendix we will work with a DAG $\mathcal{G}$ with vertex set $\mathcal{V}$ and edge set $\mathcal{E}$. The vertices will be labelled by sequential integers t which take values from 1 to the number of vertices $N = |\mathcal{V}|$. The directed edges are defined to run from the lower value vertex to the higher value vertex, so if $(s,t) \in \mathcal{E}$ then $s < t$.

A.1 Definition of reverse greedy paths

Every vertex t in a DAG $\mathcal{G}$ has two reverse greedy paths: the FORWARD REVERSE GREEDY PATH to nodes of times later than t, the other the REVERSE GREEDY PATH coming from nodes of earlier times. Here we will define the reverse greedy path as that is what is used in this work.

The reverse greedy path arriving at a vertex t is defined by following the edge arriving at that vertex t which links back to the most recent predecessor vertex s. More formally, suppose $\mathcal{N}^{(-)}(t)$ is the set of predecessors of a vertex t, the source vertices of edges whose target vertex is t:

$$\mathcal{N}^{(-)}(t) = \{s|(s,t) \in \mathcal{E}\} \tag{A.1}$$

where $\mathcal{E}$ is the edge set of the DAG. Then the reverse greedy path to vertex t is a sequence of vertices denoted as $P^{(-)}(t)$ which is defined to be

$$\begin{aligned} P^{(-)}(t) &= \{s_i | s_i = \max(\mathcal{N}^{(-)}(s_{i+1})), \, i \in \{0, \ldots, \ell(t)\}, \\ &\qquad s_0 = 1, \, s_{\ell(t)} = t\} \, . \end{aligned} \tag{A.2}$$

Note that the length of the reverse greedy path to t is denoted $\ell(t)$ so $\ell(t) = |P(t)| - 1$. The reverse greedy path will always terminate at a global SOURCE NODE, the only node with zero in-degree.

A.2 Price Model Definition

The Price model [1] (see also sec.14.1 of [2]) is a growing network model giving us a sequence of graphs $\{\mathcal{G}(t)\}$ where these are indexed by an integer t, the 'time'. Each graph has a vertex set $\mathcal{V}(t)$ and an edge set $\mathcal{E}(t)$. To create the next graph $\mathcal{G}(t+1)$ in the Price model sequence, we add one new vertex at time $(t+1)$ to $\mathcal{V}(t)$. We will overload our notation so that the positive integer t is both the 'birth' time of a vertex and it is also used to represent that vertex. So formally $\mathcal{V}(t+1) = \mathcal{V}(t) \cup \{(t+1)\}$. We choose to index our nodes from one upwards so that at time t the total number of nodes in the graph $\mathcal{G}(t)$, denoted as $N(t)$, is $N(t) = |\mathcal{V}(t)| = t$.

A new node $(t+1)$ is connected by m new edges to existing vertices s chosen with probability $\Pi(t,s)$. The number of edges in the edge set $\mathcal{E}(t)$ of the DAG $\mathcal{G}(t)$, edges between nodes created at time t or earlier, is $E(t) = E_0 + mt = |\mathcal{E}(t)|$ where E_0 is some constant. Note this is the total number of edges after we have finished adding all the incoming edges to vertex, t.

Finally the node created at time s in the graph $\mathcal{G}(t)$ has out-degree $k^{(\mathrm{out})}(t,s)$.

The connection of edges to new node $(t+1)$ as encoded by $\Pi(t,s)$ is made in one of two ways. With probability p the node $(t+1)$ is connected to an existing vertex s chosen with cumulative advantage $k^{(\mathrm{out})}(t,s)/E(t)$. Otherwise, so with probability $\bar{p} = (1-p)$, we choose an existing vertex s uniformly at random from the set of existing vertices, i.e. with probability $1/N(t)$. That is the probability of connecting new vertex $(t+1)$ to existing vertex s is*

$$\begin{aligned}\Pi(t,s) &= p\frac{k^{(\mathrm{out})}(t,s)}{E(t)} + \bar{p}\frac{1}{N(t)} \quad \text{if } t \geq s \geq 1 \ \&\ t,s \neq 1\,,\\ &= 1 \text{ if } t = s = 1\,,\\ &= 0 \text{ otherwise}\,. \end{aligned} \tag{A.3}$$

The usual form, the first equation, leaves us, however, with a problem for $\Pi(t=1, s=1)$ when looking at the attachment to the second vertex, $t=2$, if we think of the initial vertex at $t=1$ as having no incoming edges. The solution is to insist that $\Pi(t=1,s=1)=1$. We will see that this fixes the cumulative probability $\Pi_{\leq}$ to have a consistent value which is in fact all we need for this calculation.

For simplicity we will almost always assume that $E(t) = mt$ and $N(t) = t$. This is impossible to satisfy at early times as at the end of time step 1 we have one node which cannot have the m edges required since self-loops are not allowed for a DAG. In turn, this early time issue is a clear signal that there are some initial graph effects in the Price model. In practice, we can deal with this issue in various ways. If multiple edges between pairs of vertices are allowed then we can enforce this E and N constraint from step $t=2$ onwards (set $2m$ edges from node 1 to node 2) but at the cost of having a very skewed initial degree distribution. Without multiple edges, then the earliest point where we can have our $E(t) = mt$ and $N(t) = t$ is at $t = 2m+1$ if at that time we have a complete directed graph in which the first $2m+1$ nodes have edges leaving them to connect to every later node, i.e. where the edge set is $\mathcal{E}(t=2m+1) = \{(i,j)|1 \leq i < j \leq 2m+1\}$. Unless otherwise stated, we used this complete graph graph at time $t = 2m+1$ as the starting point for our numerical simulations.

We will need the cumulative probability function which is the probability that you attach all m edges from the node created at time $(t+1)$ to a node created at time s or earlier

$$\Pi_{\leq}(t,s) = \sum_{r=1}^{s} \Pi(t,r) \ \text{ if } \ t \geq s \geq 1\,, \tag{A.4}$$

$$= 0 \text{ otherwise.} \tag{A.5}$$

It is clear that we must have

$$\Pi_{\leq}(t, s=t) = 1\,, \qquad t \geq 1 \tag{A.6}$$

as all existing vertices are then of time t or less. This shows that if $\Pi_{\leq}(t=1,s=1)=1$ then we need $\Pi_{\leq}(t=1,s=1) = \Pi(t=1,s=1) = 1$ for consistency.

To find this cumulative advantage probability $\Pi_{\leq}(t,s)$ we can work in terms of an effective node, a single 'super' node, which represents the s nodes created from the initial time to time s. If we coarse grain these nodes from $t=1$ to s into one super node, then we should attach all their edges to this one super node (we can picture this as having multiple self-loops) so that algebraically the degree of this super node, $k^{(\mathrm{out})}_{\mathrm{eff}}(t,s)$, is the sum of all the degrees of the individual nodes created at or before s,

$$\begin{aligned} k^{(\mathrm{out})}_{\mathrm{eff}}(t,s) &= \sum_{r=1}^{s} k^{(\mathrm{out})}(t,r) \text{ if } t > s\,,\\ k^{(\mathrm{out})}_{\mathrm{eff}}(s,s) &= E(s) = m\,s\,. \end{aligned} \tag{A.7}$$

*In Newman's notation (equation (14.1), sec.14.1 of [2]) $\Pi(t,s) = (k^{(\mathrm{out})}(t,s)+a)/(t(c+a))$ with $a = m\bar{p}/p$ and $c = m$ (with $c + a = m/p$).

Now we can use this to write the cumulative probability for attachment as

$$\Pi_{\leq}(t,s) = \sum_{r=1}^{s} \left(p \frac{k^{(\mathrm{out})}(t,r)}{mt} + \bar{p}\frac{1}{t} \right) \tag{A.8}$$

$$= \frac{p}{mt} \left(k_{\mathrm{eff}}^{(\mathrm{out})}(t,s) + \frac{m\bar{p}}{p} s \right) \tag{A.9}$$

The master equation for this effective node is therefore

$$\begin{aligned} k_{\mathrm{eff}}^{(\mathrm{out})}(t+1,s) - k_{\mathrm{eff}}^{(\mathrm{out})}(t,s) &= \sum_{r=1}^{s} m\Pi(t,s) \\ &= m\Pi_{\leq}(t,s)\,. \end{aligned} \tag{A.10}$$

We can rewrite this in terms of a function $y(t,s)$ where

$$y(t,s) := k_{\mathrm{eff}}^{(\mathrm{out})}(t,s) + \frac{m\bar{p}s}{p}\,, \text{ with } y(s,s) = \frac{ms}{p}\,. \tag{A.11}$$

The master equation then becomes

$$y(t+1,s) - y(t,s) = \frac{p}{t} y(t,s) \tag{A.12}$$

$$\Rightarrow \quad y(t,s) = \frac{\Gamma(p+t)}{\Gamma(p+s)} \frac{\Gamma(s)}{\Gamma(t)} \frac{ms}{p} \tag{A.13}$$

using $y(s,s)$ value in (A.11). This gives

$$\Pi_{\leq}(t,s) = \frac{\Gamma(p+t)}{\Gamma(t+1)} \frac{\Gamma(s+1)}{\Gamma(p+s)}\,. \tag{A.14}$$

We note that this is separable with

$$\Pi_{\leq}(t,s) = \frac{j(s)}{j(t)}\,, \qquad j(s) = \frac{\Gamma(s+1)}{\Gamma(p+s)}\,. \tag{A.15}$$

For large t and s we have that

$$\Pi_{\leq}(t,s) \approx \left(\frac{s}{t}\right)^{\bar{p}}, \quad t,s \gg 1\,. \tag{A.16}$$

A.3 Master Equation for Reverse Greedy Path Length ℓ

We are interested in looking at the reverse greedy path in our growing network model. For this we need to know $\Pi_{\max}(t,s)$, the probability that of the m predecessor nodes connected to a new node at $(t+1)$, the oldest of them is $s = \max(\mathcal{N}^{(-)}(t+1))$. The probability that we connect a new node $(t+1)$ to nodes of age s or less is simply $(\Pi_{\leq}(t,s))^m$ where $\Pi_{\leq}(t,s)$ is the cumulative probability of attachment of (A.5). This gives us that

$$\Pi_{\max}(t,s) = (\Pi_{\leq}(t,s))^m - (\Pi_{\leq}(t,s-1))^m \quad \text{for } t \geq s \geq 1\,. \tag{A.17}$$

Note the case $s=1$ is covered as we defined $\Pi_{\leq}(t,0) = 0$. We may check that

$$\sum_{s=1}^{t} \Pi_{\max}(t,s) = (\Pi_{\leq}(t,t))^m = 1 \tag{A.18}$$

where we use the definition $\Pi_{\leq}(t,0) = 0$ and (A.6).

Let the probability that the length of the reverse greedy path, ℓ, from new node $(t+1)$ to the initial node at $t=1$, be $P(\ell,t)$. This will satisfy the equation

$$P(\ell,t+1) \;=\; \sum_{s=1}^{t} P(\ell-1,s)\Pi_{\max}(t,s)\,. \tag{A.19}$$

We can rewrite this master equation for $P(\ell,t)$ in terms of the generating function $G(z,t)$ defined as

$$G(z,t) = \sum_{\ell=0}^{\infty} z^{\ell} P(\ell,t)\,, \tag{A.20}$$

where

$$G(z=1,t) \;=\; 1 \tag{A.21}$$

$$G(z,t=1) \;=\; P(\ell=0,1) = 1\,. \tag{A.22}$$

This last follows because the only source node is the initial node at $t=1$ with reverse greedy path length zero, $\ell(t=1)=0$. Ultimately, we want to look at the average length of the reverse greedy path, $\langle \ell(t) \rangle$, which is obtained as

$$\left.\frac{\partial G(z=1,t)}{\partial z}\right|_{z=1} \;=\; \langle \ell(t) \rangle\,. \tag{A.23}$$

In terms of the generating function, the master equation (A.19) becomes

$$G(z,t+1) \;=\; \sum_{s=1}^{t} zG(z,s)\Pi_{\max}(t,s)\,. \tag{A.24}$$

Peeling off the top term of the sum in (A.24) gives us

$$\begin{aligned} G(z,t+1) \;=\;& zG(z,t)\Pi_{\max}(t,t) \\ & + \sum_{s=1}^{t-1} zG(z,s)\Pi_{\max}(t,s)\,. \end{aligned} \tag{A.25}$$

For now, we will simply assume that cumulative probability $\Pi_{\leq}(t,s)$ is separable, i.e. $\Pi_{\leq}(t,s) = h(t)j(s)$ for some functions $h(t)$ and $j(s)$. We can use the fact that $\Pi_{\leq}(t,s=t)=1$ from (A.6) to see that $h(t) = 1/j(t)$ so that $\Pi_{\leq}(t,s) = j(s)/j(t)$. We have the precise form for $j(s)$ in the Price model in (A.15). That gives us that $\Pi_{\max}(t,s)$ is also separable and this can be written as

$$\Pi_{\max}(t,s) \;:=\; \frac{H(s)-H(s-1)}{H(t)} \tag{A.26}$$

$$H(t) \;=\; (j(t))^{m}\,. \tag{A.27}$$

Then

$$\begin{aligned} G(z,t+1) = {}& zG(z,t)\Pi_{\max}(t,t) \\ & + \sum_{s=1}^{t-1} zG(z,s)\frac{H(s)-H(s-1)}{H(t)} \end{aligned} \tag{A.28}$$

$$\begin{aligned} =\;& zG(z,t)\Pi_{\max}(t,t) \\ & + \frac{H(t-1)}{H(t)} \sum_{s=1}^{t-1} zG(z,s)\Pi_{\max}(t-1,s)\,. \end{aligned} \tag{A.29}$$

Using (A.24) leaves us with

$$G(z,t+1) = \left(z\Pi_{\max}(t,t) + \frac{H(t-1)}{H(t)}\right) G(z,t) \tag{A.30}$$

$$= \left(z + (1-z)\frac{H(t-1)}{H(t)}\right) G(z,t)\,, \tag{A.31}$$

which has the formal solution

$$G(z,t) = \prod_{s=1}^{t-1} \left(z + (1-z)\frac{H(s-1)}{H(s)}\right) \tag{A.32}$$

given $G(z,1) = 1$. Now we can check $z = 1$ value which is clearly $G(z=1,t) = 1$ as required.

We can obtain the precise form for the Price model by using the form for $H(s)$ in (A.27) given $j(s)$ in the Price model from (A.15). This gives us that

$$\frac{H(s-1)}{H(s)} = \left(\frac{s-\bar{p}}{s}\right)^m . \tag{A.33}$$

The solution for the generating function then becomes

$$G(z,t) = \prod_{s=1}^{t-1} \left(z\left(1 - \left(\frac{s-\bar{p}}{s}\right)^m\right) + \left(\frac{(s-\bar{p})}{s}\right)^m \right) . \tag{A.34}$$

In principle this is a ratio of m-th order polynomials in s so you can write this product as the product of m ratios of Gamma functions, one term per root of the polynomial.

Average Path Length

We can find $\ell(t)$, the average length of the reverse greedy path in the Price model, from this general solution (A.30) using (A.23) and $G(z=1,t)$ from (A.21). This gives us that

$$\frac{\partial}{\partial z} G(z,t) = \sum_{s=1}^{t-1} \left[\left(1 - \frac{H(s-1)}{H(s)}\right) \prod_{r=1, r\neq s}^{t-1} \left(z + (1-z)\frac{H(r-1)}{H(r)}\right) \right] \tag{A.35}$$

$$= \sum_{s=1}^{t-1} \left[\left(\frac{1 - (H(s-1)/H(s))}{z + (1-z)(H(s-1)/H(s))} \right) \prod_{r=1}^{t-1} \left(z + (1-z)\frac{H(r-1)}{H(r)}\right) \right] \tag{A.36}$$

$$= \left[\sum_{s=1}^{t-1} \left(\frac{H(s) - H(s-1)}{zH(s) + (1-z)H(s-1)} \right) \right] G(z,t) \tag{A.37}$$

so then

$$\langle \ell(t) \rangle = \left. \frac{\partial}{\partial z} G(z,t) \right|_{z=1} \tag{A.38}$$

$$= \left[\sum_{s=1}^{t-1} \left(\frac{H(s) - H(s-1)}{H(s)} \right) \right] G(z=1,t) \tag{A.39}$$

and finally

$$\langle \ell(t) \rangle = \sum_{s=1}^{t-1} \left(1 - \frac{H(s-1)}{H(s)}\right) \tag{A.40}$$

For the Price model we have $H(s)$ from (A.33) and so we find that

$$\langle \ell(t) \rangle = \sum_{s=1}^{t-1} \left(1 - \left(\frac{s-\bar{p}}{s} \right)^m \right) \tag{A.41}$$

Now we can expand the product using the binomial expansion to find that

$$\langle \ell(t) \rangle = \sum_{n=1}^{m} \binom{m}{n} (-1)^{n-1} (\bar{p})^n \sum_{s=1}^{t-1} s^{-n} \tag{A.42}$$

For each value of n we can write the s^{-n} series as the difference of two Hurwitz zeta functions $\zeta(m,t)$

$$\langle \ell(t) \rangle = \sum_{n=1}^{m} \binom{m}{n} (-1)^{n-1} (\bar{p})^n \left(\zeta(n,1) - \zeta(n,t) \right) . \tag{A.43}$$

The Hurwitz zeta functions are finite for $t > 0$ and $n > 1$ so we see that the terms $n \geq 2$ only contribute a constant plus terms from $\zeta(n,t)$ which fall off as t^{1-n} or faster.

The leading term in the large time limit of $\langle \ell(t) \rangle$ comes only from the $n = 1$ term in (A.42) and this is

$$\langle \ell(t) \rangle_{n=1} \approx m\bar{p} \ln(t) - m\bar{p}\psi(m\bar{p}+1) + O(t^{-1}) \tag{A.44}$$

where $\psi(t)$ is the digamma function.

The constant in an asymptotic expansion in t of $\langle \ell(t) \rangle$ picks up further contributions from the $n \geq 2$ terms so we have that

$$\begin{aligned} \lim_{t\to\infty} \langle \ell(t) \rangle &= m\bar{p} \ln(t) - m\bar{p}\psi(m\bar{p}+1) \\ &+ \sum_{n=2}^{m} \binom{m}{n} (-1)^{n-1} (\bar{p})^n \zeta(n) + O(t^{-1}) \end{aligned} \tag{A.45}$$

where it is implicit that there is no contribution from the term with the sum for the case of $m = 1$. Here $\zeta(n)$ is the Riemann zeta-function.

Variance

We can also use the generating function solution (A.34) to find the variance $\sigma^2(t)$ of the reverse greedy path length. We have that

$$\langle \ell(t)(\ell(t)-1) \rangle = \left. \frac{\partial^2}{\partial z^2} G(z,t) \right|_{z=1} \tag{A.46}$$

We have that

$$\begin{aligned} \frac{\partial^2}{\partial z^2} G(z,t) &= \left[\left(\sum_{s=1}^{t-1} \frac{H(s)-H(s-1)}{zH(s)+(1-z)H(s-1)} \right)^2 \right. \\ & \left. - \left(\sum_{s=1}^{t-1} \left(\frac{(H(s)-H(s-1))^2}{(zH(s)+(1-z)H(s-1))^2} \right) \right) \right] G(z,t) \end{aligned} \tag{A.47}$$

so that

$$\langle \ell(t)(\ell(t)-1)\rangle = \left[\left(\sum_{s=1}^{t-1}\frac{H(s)-H(s-1)}{H(s)}\right)^2 - \left(\sum_{s=1}^{t-1}\left(\frac{(H(s)-H(s-1))^2}{(H(s))^2}\right)\right)\right] \quad (A.48)$$

$$= (\langle \ell\rangle)^2 - \left(\sum_{s=1}^{t-1}\left(\frac{(H(s)-H(s-1))^2}{(H(s))^2}\right)\right) \quad (A.49)$$

With $\sigma^2(t) = \langle(\ell(t))^2\rangle - (\langle \ell(t)\rangle)^2$ we have that

$$\sigma^2(t) - \langle \ell(t)\rangle = \langle \ell(t)(\ell(t)-1)\rangle - (\langle \ell(t)\rangle)^2 \quad (A.50)$$

$$= -\sum_{s=1}^{t-1}\left(1-\frac{H(s-1)}{H(s)}\right)^2 \quad (A.51)$$

$$= -\sum_{s=1}^{t-1}\left(1-\left(\frac{(s-\bar{p})}{s}\right)^m\right)^2 \quad (A.52)$$

$$= -(m\bar{p})^2\sum_{s=1}^{t-1}(s)^{-2} + O(m^3\bar{p}^3) \quad (A.53)$$

In the long-time limit we have that

$$\lim_{t\to\infty}\sigma^2(t) - \langle \ell(t)\rangle = -\frac{\pi^2}{6}m^2(\bar{p})^2 + O(m^3\bar{p}^3) \quad (A.54)$$

Note that these correction are all finite so we have basically shown that the variance grows with the mean i.e. Poisson-like behaviour.

Long-time Generating Function Result

From (A.34) the logarithm of the generating function $G(z,t)$

$$\ln(G(z,t)) = \sum_{s=1}^{t-1}\ln\left(1+(z-1)\left(1-\left(1-\frac{\bar{p}}{s}\right)^m\right)\right). \quad (A.55)$$

Expanding around $z=1$, or with $\bar{p}$ small, we have that

$$\ln(G(z,t)) = -\sum_{q=1}^{\infty}\frac{(1-z)^q}{q}\sum_{s=1}^{t-1}\left(1-\left(1-\frac{\bar{p}}{s}\right)^m\right)^q. \quad (A.56)$$

We recognise the first term as $\langle \ell(t)\rangle$ from (A.41) so that

$$\ln(G(z,t)) = (z-1)\langle \ell(t)\rangle - \frac{(1-z)^2}{2}\sum_{s=1}^{t-1}\left(\frac{(m\bar{p})^2}{s^2}+\ldots+\frac{\bar{p}^{2m}}{s^{2m}}\right)$$
$$-\sum_{q=3}^{\infty}\frac{(1-z)^q}{q}\sum_{s=1}^{t-1}\left(1-\left(1-\frac{\bar{p}}{s}\right)^m\right)^q \quad (A.57)$$

$$= (z-1)\langle \ell(t)\rangle - \frac{(1-z)^2}{2}\bar{p}^2\left(m^2\zeta(2)+\ldots+\zeta(2m)\bar{p}^{2(m-1)}\right)$$
$$-O(\bar{p}^3(1-z)^3). \quad (A.58)$$

That is the leading term comes from the only term with a sum over $1/s$ which diverges as $\ln(t)$ and is completely captured by the $\langle \ell(t) \rangle$ contribution. The higher order terms in an expansion around $z = 1$ are all finite which may be expressed as polynomials in $\bar{p}$ with terms from $\bar{p}^q$ to $\bar{p}^{qm}$ for the coefficient of the $(1-z)^q$ term.

From this we wee that the leading order term in $G(z,t)$ in the long time limit

$$\lim_{t\to\infty} G(z,t)) \quad = \quad \exp((z-1)\langle \ell(t) \rangle) \tag{A.59}$$

which is the generating function for a Poisson distribution. So we deduce that in the long-time limit, the distribution of lengths of the reverse greedy path in the Price model is a Poisson distribution with mean equal to $m\bar{p}\ln(t)$.

$$\lim_{t\to\infty} P(\ell,t) = \frac{e^{-\lambda}\lambda^\ell}{\ell!} \qquad \lambda = \langle \ell(t) \rangle \approx m\bar{p}\ln(t)\,. \tag{A.60}$$

A.4 Reverse Greedy Path for $m = 1$

The Price model for $m = 1$ is a special case in this model as then the graphs produced are directed trees. Various aspects of this $m = 1$ case of the Price model and its variants can be studied analytically, for example [3, 4]. Here we just note that many of the equations in our calculations become simple first order polynomials, such as for $\Pi_{\max}(t,s)$ in (A.17), which allows for a simple direct solution in the $m = 1$ that case. The solutions for the reverse greedy path length also takes on a much simpler form.

For $m = 1$ we have from (A.34) that the generating function is just

$$G(z,t;m=1) \quad = \quad \prod_{s=1}^{t-1} \frac{(s+(z-1)\bar{p})}{s} = \frac{\Gamma(t+(z-1)\bar{p})}{\Gamma(1+(z-1)\bar{p})\Gamma(t)}\,. \tag{A.61}$$

Similarly, the expression for the reverse greedy path length is also simple:

$$\langle \ell(t) \rangle \quad = \quad \sum_{s=1}^{t-1} \frac{\bar{p}}{s} = \bar{p}(\psi(t) - \gamma) \tag{A.62}$$

where $\gamma \approx 0.577$ is the Euler-Mascheroni constant.

A.5 Vertex Partitions

A partition $\mathcal{P}$ of the vertex set $\mathcal{V}$ is a set of non-overlapping non-empty subsets which contain each and every vertex once and only once. That is $\mathcal{P} = \{\mathcal{P}(i)\}$ where the BLOCKS of the partition, $\mathcal{P}(i)$, are such that $\mathcal{P}(i) \subset \mathcal{V}$, $\mathcal{P}(i) \neq \emptyset$, $\mathcal{P}(i) \cap \mathcal{P}(j) = \emptyset$ unless $i = j$, and $\cup_i \mathcal{P}(i) = \mathcal{V}$. The definition of a unique integer length scale associated with each node in any one instance of the model gives a natural partition of the set of vertices. reverse greedy path to each node t from a source node, $\ell(t)$.

Of particular interest here is a partition in terms of the longest path $L(t)$ to a given node t. In any DAG, the HEIGHT of a node is the length of the longest path to a node from any global source node, any node with zero in-degree. Thus in the Price model, the height of a node is simply the longest path length from the initial node to any node, that is our $L(t)$ is the height. A natural partition of the set of vertices in a DAG is where each block, $\mathcal{P}_{\text{height}}(h)$, contains all the vertices of height h,

$$\mathcal{P}_{\text{height}}(h) = \{t \,|\, t \in \mathcal{V},\, L(t) = h\}\,, \quad h \in \mathbb{Z}\,. \tag{A.63}$$

This partition by height has the special property that no two vertices in any one block are connected by any path. In a DAG nodes connected by a path cannot be of the same height. A set of disconnected vertices is known as an ANTICHAIN so the blocks of the height partition are all antichains. In some sense, the vertices in an antichain can be considered to be of equivalent ages. There is no relationship between them which says that any one vertex in the antichain need to come before or after another. The question here is can we estimate some of the properties of these height antichains? To do that we need to define a slightly different partition.

For the Price model, we can also define another type of partition in terms of the average path length $\langle \ell(t) \rangle$ associated with a node created at time t averaged over all possible instances of the Price model. We will discuss this partition in terms of the reverse greedy path length ℓ where we have analytical results but the construction is identical for the longest path length L. Since our average path lengths are monotonically increasing functions of time t, we can define the blocks $\widehat{\mathcal{P}}(\hat{\ell})$ of one partition in terms of an integer value $\hat{\ell} \in \mathbb{Z}$ as follows. All nodes in one block of the partition have an expectation value for their reverse greedy path length which rounds to the integer $\hat{\ell}$,

$$\widehat{\mathcal{P}}(\hat{\ell}) = \{t \,|\, t \in \mathcal{V},\, \hat{\ell} - \frac{1}{2} \leq \langle \ell(t) \rangle < \hat{\ell} + \frac{1}{2},\, \hat{\ell} \in \mathbb{Z}^+\}\,. \tag{A.64}$$

The idea here is that we can estimate the properties of the blocks in this partition, $\widehat{\mathcal{P}}(\hat{\ell})$, by using the leading behaviour for the lengths scales, namely they grow logarithmically with t. We would then expect that the partitions of a single instance based on the measured values of an integer valued path length would show similar features.

The leading behaviour for the mean reverse greedy path length of a node t can be expressed as

$$t = \tilde{t}(\mu_{\mathrm{gr}})^{\bar{\ell}} \tag{A.65}$$

where $\bar{\ell} = \langle \ell(t) \rangle$ while μ_{gr} and $\tilde{t}$ are some constants. Our leading order results give $\mu_{\mathrm{gr}} = \exp(1/(m\bar{p}))$ for the reverse greedy path, while we can estimate $\mu = \exp(1/a)$ and $\tilde{t} = \exp(-b/a)$ using the numerical results for the coefficients a and b obtained from the fit to $a\ln(t) + b$.

From this behaviour we can estimate various properties of the blocks $\widehat{\mathcal{P}}(\hat{\ell})$. It is useful to express these results in terms of the time scale $\hat{t}(\hat{\ell})$ (not necessarily an integer) for a node whose path length is expected to be the integer $\hat{\ell}$, namely $\hat{t}(\hat{\ell}) = \tilde{t}(\mu_{\mathrm{gr}})^{\hat{\ell}}$ for $\hat{\ell} \in \mathbb{Z}^+$. Other properties of the path length partition $\widehat{\mathcal{P}}(\hat{\ell})$ can be estimated in terms of $\hat{t}$: the average time of nodes in the partition $\bar{t}(\hat{\ell})$, the standard deviation of the times of nodes in the partition $\sigma_t(\hat{\ell})$ and the number of nodes in the block $|\widehat{\mathcal{P}}|(\hat{\ell})$. We find that

$$\bar{t}(\bar{\ell}) = \cosh(\sqrt{\mu_{\mathrm{gr}}})\, \hat{t}(\bar{\ell}) \tag{A.66}$$

$$\sigma_t(\bar{\ell}) \approx \frac{1}{\sqrt{3}} \sinh(\sqrt{\mu_{\mathrm{gr}}})\, \hat{t}(\bar{\ell}) \tag{A.67}$$

$$|\widehat{\mathcal{P}}|(\bar{\ell}) = 2\sinh(\sqrt{\mu_{\mathrm{gr}}})\, \hat{t}(\bar{\ell}) \tag{A.68}$$

The results above are for the partition into blocks $\widehat{\mathcal{P}}(\hat{\ell})$ of (A.64) which are defined in terms of averages over an ensemble. More interesting is to look at the partition into height antichains, $\mathcal{P}_{\mathrm{height}}(h)$ of (A.63) since this based on the topology of a single network and so much more relevant to studies of real data sets. Our suggestion is that in the Price model all the length scales behave in the same way, following our description in (A.65). So we should expect that all partitions in the Price model based on such length scales should show the scaling behaviour suggested in (A.68). For example that suggests that for the height antichains, we should compare the ratios of a particular property for adjacent blocks, for instance $\bar{t}(h+1)/\bar{t}(h)$, since this analysis suggests such ratios will be constant.

B Numerical Implementation

B.1 Price Model Algorithm

To reduce the number of random numbers drawn, we use the following algorithm. First we observe that the probability $\Pi(t, s)$ of (A.3) used to choose existing nodes s to attach to a new node $(t+1)$ is proportional to $\Pi(t, s) \propto k^{(\text{out})}(t, s) + (m\bar{p}/p)$. If we limit ourselves to parameter values where $\alpha = (m\bar{p}/p)$ is a non-negative integer, then we can choose existing vertices with probability $\Pi(t, s)$ by drawing uniformly at random from a list, `attachment_list`, if it is properly formed. We define our algorithm as follows.

1. Set initial graph `G` with t nodes.
2. Initialise `attachment_list` to match initial graph.
   - For every edge in the initial graph, we add the source end of the edge to `attachment_list`. That is is $(s, t) \in \mathcal{E}(t)$ where $s < t$ then we add s only to `attachment_list`.
   - Each node in the initial graph is added $\alpha = (m\bar{p}/p)$ times to `attachment_list`.
3. Increment t.
4. Draw m times uniformly at random from `attachment_list`, to give the list (sequence) $S(t) = [s_1, s_2, \ldots, s_m]$.
   - If drawn with replacement, this is simple and fast and it matches the assumptions implicit in the algebraic calculations. This allows multiple edges between nodes.
   - If drawn without replacement we avoid multiple edges but this is liable to be slower. However this constraint is not enforced in the analytic equations.
5. Append the m nodes in the list $S(t)$ to `attachment_list`.
   This captures the cumulative advantage process.
6. Append $\alpha = (m\bar{p}/p)$ copies of t to `attachment_list`.
   This encodes the uniform random process.
7. If adding more nodes, return to step 3.

We allowed multiple edges in our simulations and in this approach one random number was needed for every edge added, a total of mN random numbers if the final network had N nodes. For the attachment list $(m + \alpha)N$ memory locations are needed. Note that in our work here we only created the `attachment_list` along with lists for the length of paths to each vertex. There was no need for use to create the full network structure. However, should the full network structure be required, it can be deduced later from `attachment_list` as defined here.

Note that the $p = 0$ case is treated separately since the attachment list is not needed. In that case we draw uniformally at random from the set of nodes, with replacement in our case since we allow multiple edges.

In our work we choose to allow multiple edges in the networks created in our simulations. This will have relatively little effect on results as the model is inherently a sparse graph model. As the number of nodes increases, as m is fixed, the chance of choosing the same existing vertex twice when connecting it to a new node decreases.

As noted elsewhere, our initial graph was a DAG of $2m + 1$ nodes with an edge between every node, that is the initial edge set was $\mathcal{E}(2m + 1) = \{(s, t)|1 \leq s < t \leq 2m + 1\}$. This has $E(t) = mt$ for $t \geq 2m + 1$. However, we did investigate the effect of the initial graph and this is discussed in Section B.4.

An alternative algorithm for the Price model, which we did not use here, would be to carry two separate lists of existing vertices, one used for the cumulative advantage process, and one for the selecting uniformly at random from the set of vertices. This is equally easy to code. The disadvantage is that we would now need to draw an additional random number for every edge added, a total now of $2mN$, as this extra random number has to be compared against the parameter p in order to decide which list to draw from. The extra random numbers would slow the process but the gain would be flexibility as there would be no restriction on the parameters to ensure that $\alpha = (m\bar{p}/p)$ was integer. This alternative method would also only require $(m+1)N$ memory locations for the lists.

B.2 Numerical Reverse Greedy Path Algorithm

Numerically, the reverse greedy path may be found as follows

1. Set initial graph `G` with t nodes.
2. Set the reverse greedy path length for the initial t nodes, `greedy_length[s]` for $s = 1, 2, \ldots, t$.
3. Set the nearest neighbour along this reverse greedy path length for the initial t nodes, `greedy_neighbour[s]` for $s = 1, 2, \ldots, t$.
4. Increment t.
5. Add a new node index t to `G`.
6. Update graph `G` by adding m new edges, from vertices $\{s\}$ to new node t, which then define the past neighbour set of vertex t, i.e. $\mathcal{N}^{(-)} = \{s\}$.
7. Find the past neighbour with the largest time $s_{\text{gr}} = \max(\mathcal{N}^{(-)}(t))$.
8. Record this closest past neighbour as the last step on the reverse greedy path to t, i.e. set `greedy_neighbour[t]` $= s_{\text{gr}}$.
9. Set the length of the reverse greedy path of new node t to be one more than the length of the reverse greedy path to this nearest past neighbour s_{gr}, so set $\ell(t) = \ell(s_{\text{gr}}) + 1$. Numerically we just need to set
   `greedy_length[t]=`

   `greedy_length[greedy_neighbour[t]]+1`.
10. If adding more nodes, return to step 4.

The length of the reverse greedy path from the first node $t = 1$ to a given node t is now stored for each node. The vertices on the unique reverse greedy path can be found by iterating back though the `greedy_neighbour` property of the nodes. If the specific path used is not required we need not record this information.

We also note that the length of the longest path, the longest path from the initial node $t = 1$ to any node t, may be tracked numerically in a similar way. Such a longest path is always exists but is not unique so we could only ever record one example longest path using this approach.

B.3 Fitting

For each set of parameters p, m, N, we ran the model $R = 100$ times. We collected the length of the reverse greedy path and of the longest path for each node created at time t. In this case, each data point y_{tr} is the relevant path length, $\ell(t)$ or $L(t)$, measured for node t on run r.

We make several assumptions about our data. First, we assume that the measurements we make on each individual run are independent. This assumption is not completely true in our data. Secondly, we assume that the set of measurements at the same point t across many runs have a distribution that is normally distributed. As Fig. B1 shows, this is approximately true.

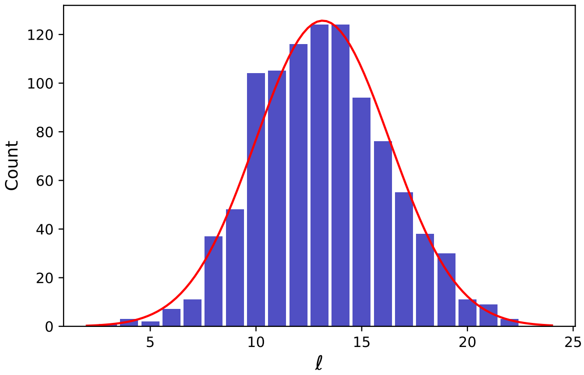

(a) Greedy path lengths.

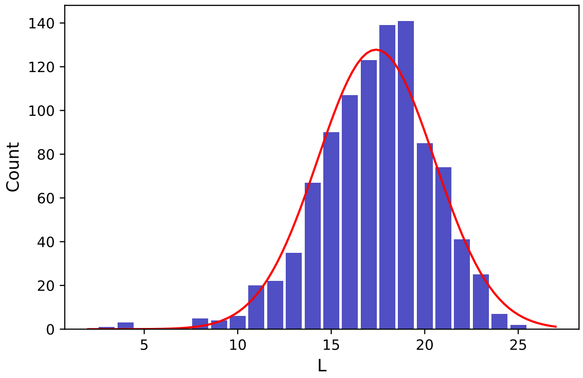

(b) The longest path lengths.

Figure B1: Distributions of the reverse greedy path length (left) and longest path lengths (right) for a node with index $t = 10^5$ in the Price model with $m = 5$, $c = 1$ and 10^6 total number of nodes, averaged over 1000 networks. For comparison, a Gaussian is plotted with the same mean and standard deviation as each data set. We can see that a normal distribution,, while not perfect, is a sufficiently good description of the distribution to justify our use of gaussian based fitting statistics.

To evaluate the quality of our fit, we looking for parameter values which minimised the value of the chi-squared function (for instance see [5])

$$\chi^2 = \sum_{t,r} \frac{(y_{tr} - f_t)^2}{(\sigma_t)^2} \tag{B.1}$$

where y_{tr} is a path length of the node created at time t as measured in run r. The fitting function used in the main text is $f_t = a\ln(t) + b$ but we also tried $f_t = a\ln(t) + b + (c/t)$ where a, b, c, are constants to be found by fitting.

For numerical convenience (minimising memory requirements), for each of the path length we kept track of two values for each node occurring at t, the total sum of values T_t and the sum of squares S_t, as given by

$$T_t = \sum_r y_{tr}, \quad S_t = \sum_r (y_{tr})^2 . \tag{B.2}$$

We do not expect the fit to be valid for small system sizes. For instance, in most of our work the initial graph is a complete DAG (see Appendix B.4 for a discussion of initial graph effects) in within this initial the path lengths scale as $y_t = L(t) = \ell(t) = t$. To deal with this we fit our data from time t_0, an additional parameter in our fit, to the largest time which is equal to the number of nodes N. So, in terms of the totals and the squares of totals, our chi-squared function becomes

$$\chi^2 = \sum_{t=t_0}^{N} \frac{S_t - 2T_t f_t + R(f_t)^2}{(S_t/R) - (T_t/R)^2} . \tag{B.3}$$

Under our assumptions of normal fluctuations and independence as noted above, the probability of obtaining a particular χ^2 value is given by the integral of the complementary cumulative distribution function of the χ^2 distribution for the given degrees of freedom. The number of degrees of freedom in the model is equal to $(N - t_0 + 1).R$. We varied the t_0 parameter between 100 and $10,000$ but found that this cutoff parameter t_0 had no significant influence on the resulting fits. This is to be expected, as even the largest t_0 value considered, $10,000$, constitutes a mere 0.1% of the data we were using. So, in our work we used a fixed value of $t_0 = 1,000$.

The errors in the parameters of the linear fit to $f_t = a\ln(t) + b$ come from the square root of the diagonal elements of the covariance matrix. We found that there was no consistent pattern in the next-to-leading order terms, that is in b, so these are not shown. As noted elsewhere, this constant factor b in our fit is likely to be effected by the form of the initial graph since this can add a significant constant to the lengths scales and the initial graph will effect lengths scales later in different ways depending on the parameters chosen. We also tried looking at higher order terms, using non-linear fits to $f(t) = a\ln(t) + b + (c/t)$, but again saw no improvements in our results. For the results of the comparison between the linear and non-linear fits see Table B1.

m	α	$\chi^2_{\mathrm{gr,lin}} - \chi^2_{\mathrm{gr,nonlin}}$	$\frac{\chi^2_{\mathrm{gr,lin}} - \chi^2_{\mathrm{gr,nonlin}}}{\chi^2_{\mathrm{gr,lin}}} \times 10^7$	$\chi^2_{\mathrm{max,lin}} - \chi^2_{\mathrm{max,nonlin}}$	$\frac{\chi^2_{\mathrm{max,lin}} - \chi^2_{\mathrm{max,nonlin}}}{\chi^2_{\mathrm{max,lin}}} \times 10^7$
2	1	1914.679	1.8958	52115.2	51.6
2	2	5500.539	5.4468	47292.36	46.9
2	3	10679.31	10.574	32425.11	32.1
2	4	15793.18	15.638	11345.38	11.2
3	1	4128.344	4.0881	53148.67	52.7
3	2	10891.1	10.784	30573.03	30.3
3	3	18021.77	17.849	14582.06	14.5
3	4	29150.27	28.865	535.4066	0.531
4	1	6381.005	6.3185	35694.73	35.4
4	2	15188.28	15.041	13707.66	13.6
4	3	30406.43	30.109	10.1752	0.0101
4	4	41108.91	40.72	3412.088	3.39
5	1	7572.032	7.4983	31364.85	31.1
5	2	23749.52	23.52	3088.395	3.06
5	3	36774.17	36.424	1128.841	1.12
5	4	66946.58	66.314	47772.77	47.3
6	1	11489.57	11.378	22020.32	21.8
6	2	26992.08	26.731	149.04	0.148
6	3	61386.35	60.782	45868.17	45.4
6	4	89429.041	88.563	93100.79	92.2

Table B1: Absolute and relative difference between chi-squared values obtained by fitting path data to a linear function, (3.1), χ^2_{lin} and to a non-linear function $f(t) = a\ln(t) + b + (c/t)$, χ^2_{nonlin}. The non-linear fit results in smaller χ^2 values, but the improvement is marginal.

B.4 Initial graph effect

Throughout this work, we started our simulations from an initial graph which was the directed complete graph composed of $(2m + 1)$ nodes. That is $\mathcal{G}_{\mathrm{compl}}(t)$ where $\mathcal{V}_{\mathrm{compl}}(t) = \{1, 2 \ldots, t\}$ and $\mathcal{E}_{\mathrm{compl}}(t) = \{(s, r) | 1 \leq s < r \leq t\}$. This is a dense, transitively complete graph but at each

step in our simulation we always have exactly m more edges than nodes which is an assumption in the analytical work. A possible drawback is that this initial graph has long path lengths, $\ell(t) = L(t) = (t-1)$ for nodes in this graph. As both our longest and reverse greedy paths will start with paths in the initial graph, these long initial graph path lengths will produce a significant addition to those we measure. For instance, for $m = 5$ this initial graph contributes up to 10 to any path we measure while the typical length scales we measure at late times are less than 100 in general.

We can, however, choose alternative initial graphs. To match the standard Price model and our analysis, we confine ourselves to initial graphs which are weakly connected and which have a single global source node[†] where $k^{(\mathrm{in})} = 0$. A transitively reduced [6] version of our chosen initial graph is a single chain of nodes, so $\mathcal{G}_{\mathrm{chain}}(t)$ where $\mathcal{V}_{\mathrm{chain}}(t) = \{1, 2 \ldots, t\}$ and $\mathcal{E}_{\mathrm{chain}}(t) = \{(r, r+1) | 1 \leq r < t\}$, would also be a valid choice. This type of initial graph is as sparse as possible, all nodes have $k^{(\mathrm{in})}$ and $k^{(\mathrm{out})}$ equal to zero or one, but this initial graph has the same long path lengths as the complete graph. The reason the chain and the complete graph lead to different behaviour in the shift in the average path length at long times is because of their different initial degree distributions which alters the pattern of attachment at early times. That in turn alters the likelihood that the paths we measure join the initial graph at a particular node. In particular, we expect that the paths we measure in models starting with the complete graph are more likely to contain a smaller fraction of the initial graph as the earliest nodes will have higher out degrees but add shorter path lengths to our measurements. By way of contrast, in the chain, most nodes have the same initial degree, they are likely to have similar degree over time so the paths we measure are more likely to leave the initial graph at a later node so giving a longer contribution to the path length coming from this initial chain graph.

Alternatively, we could also use an initial graph, in which the single global source node points to the remaining nodes, which in turn are not pairwise connected. This is a "directed star graph" $\mathcal{G}_{\mathrm{star}}(t)$ where $\mathcal{V}_{\mathrm{star}}(t) = \{1, 2 \ldots, t\}$ and $\mathcal{E}_{\mathrm{star}}(t) = \{(1, r) | 1 < r \leq t\}$. Now the degree distribution is highly skewed, with the central node going to dominate attachment through the the cumulative advantage mechanism. However, connecting to one of the other nodes only adds one to the paths we measure so we expect our models using this initial star graph will have the smallest paths lengths of the three cases considered.

As Fig. B2 shows, the results obtained using either of the three studied variants of the initial graph are distinguishable. The asymptotic scaling of the path lengths as $a \ln t$ remains the same with the same value of the parameter a. However the constant contribution, b of (3.1), seems to depend on the choice of the initial graph. As Fig. B2 and Table B2 show, the b coefficient behaves exactly as suggested above: the star graph gives the shortest path lengths while the chain gives the longest.

B.5 Next-to-leading Order Coefficient

The next-to-leading order coefficient, b of (3.1) did not reveal as clear trends as the coefficient a of the same equation. There does not seem to be an obvious relation between b_{max} and b_{gr}, as Fig. B3 shows.

[†] In fact, if we have several global source nodes we could add just one more "global parent node" with an edge from this extra node to all of the original global source nodes. This adds one to all all the lengths we measure but creates a DAG with a single node of $k^{(\mathrm{in})} = 0$.

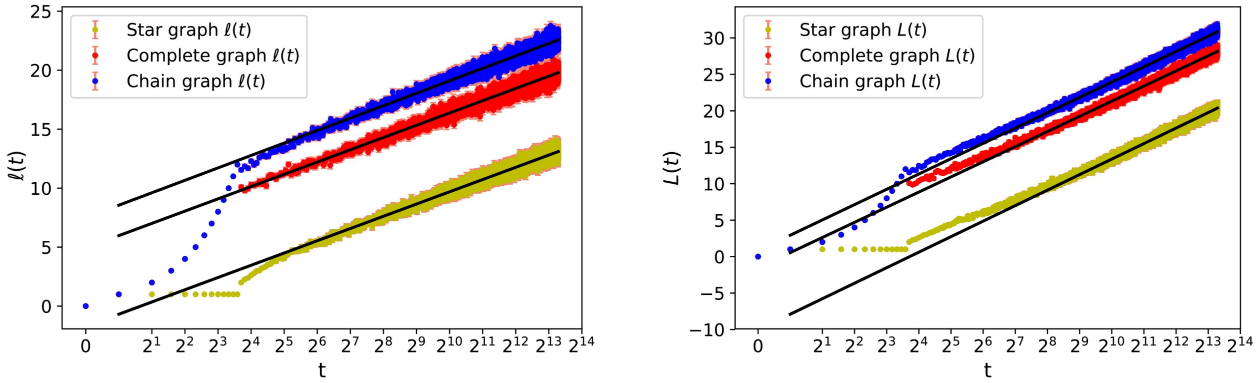

Figure B2: Path length (greedy on the left and the longest on the right) vs. $\ln(t)$ for 100 runs with m=6, fitness=2, $N = 10^5$ using different initial graphs, all composed of $2m + 1$ nodes. Fitted lines are of the form $a \ln(t) + b$. The fit parameter a seems to be unaffected by the initial graph, whereas the fit parameter b varies significantly, see Table B2. This causes the slope to remain stable but shifts the graphs vertically. The fit was obtained for nodes created from $t = 1,000$ onwards.

Path type	Initial graph	a	b
Greedy	Star	1.4973 ± 0.0052	-0.688 ± 0.04417
Greedy	Complete	1.5001 ± 0.0038	5.9741 ± 0.0309
Greedy	Chain	1.5213 ± 0.0035	8.5548 ± 0.0290
Longest	Star	3.0701 ± 0.0056	-7.9019 ± 0.0476
Longest	Complete	2.9970 ± 0.0034	0.5458 ± 0.0279
Longest	Chain	3.0303 ± 0.0034	2.9345 ± 0.02789

Table B2: Variation in obtained fitting parameters for the greedy path scaling and the longest path scaling when using various initial graphs in the Price model with $m = 6$, $\alpha = 2$. The data from $t = 1,000$ to $t = 10^5$ was fitted to the form $a \ln(t) + b$. The initial graph seems to have a small effect on the slop, parameter a, but causes significant changes in the intercept, parameter b.

B.6 Height Antichain Properties

The HEIGHT of a node in a DAG is the length of the longest path to a node from any global source node, any node with zero in-degree. Thus in the Price model, the height of a node is simply the longest path length from the initial node to any node, our L. We can also define ANTICHAINS, sets of nodes which are not connected by any path, for example see [7]. A simple example in a DAG is a HEIGHT ANTICHAINS, a set of nodes which are all at the same height since nodes connected by a path cannot be of the same height. Based on the $\ln(t)$ scaling of the reverse greedy path length, we can speculate that the average height L of a node t will scale as $t = \tilde{t}_{\max}(\mu)^L$. Then, as discussed in Section A.5, we find that various properties of the antichains should scale in a simple way with height: the average time of nodes in the partition $\bar{t}(L)$ as in (A.66), the standard deviation of the times of nodes in the partition $\sigma_t(L)$ as in (A.67), and the number of nodes in the block $|\widehat{\mathcal{P}}|(L)$ as in (A.68). Results for ratio of these quantities for adjacent blocks are shown in Fig. B4.

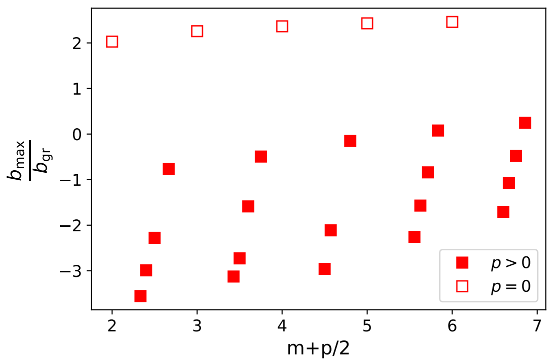

Figure B3: The ratio of $b_{\max}$ and b_{gr} where b is the next-to-leading order coefficient in the best fit of the numerical path length data to $a\ln(t) + b$: $b_{\max}$ for the longest path data and b_{gr} for the reverse greedy path data. These values were obtained by fitting the form to nodes created between $t = 1,000$ and $t = 10^8$ from 100 realisations. The errors on the fitted values of b, as estimated from the covariance matrix of the linear fitting algorithm, were smaller than the marker size so the uncertainties are not shown.

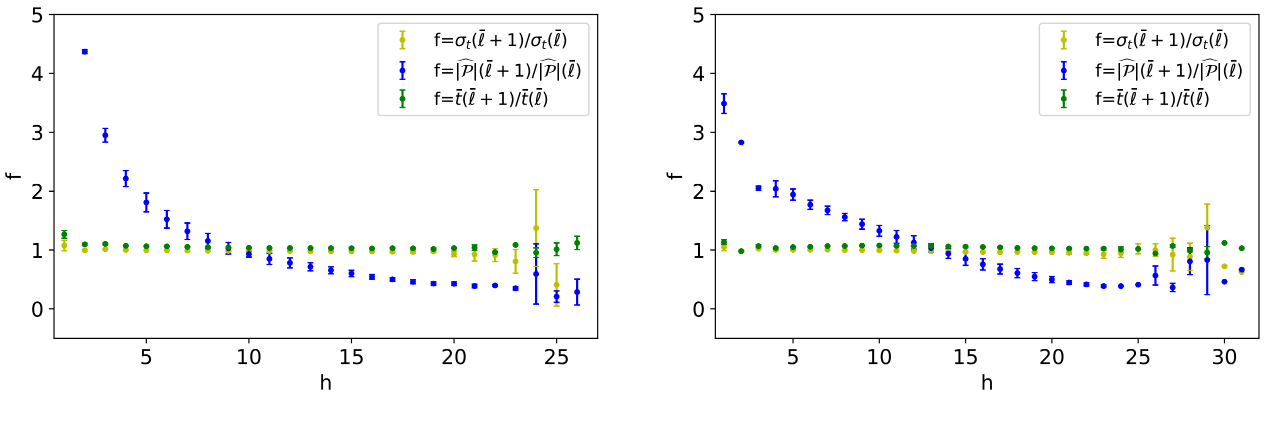

(a) Greedy path partition.

(b) The longest path partition.

Figure B4: Ratio of quantities related to vertex partitions: the average time of nodes in the partition $\bar{t}(\bar{\ell})$, the standard deviation of times $\sigma_t(\bar{\ell})$ and the number of nodes in the block for adjacent blocks $|\widehat{\mathcal{P}}|(\bar{\ell})$ for greedy path partition on the left and for the height antichains on the right.

References

[1] D. S. Price, A general theory of bibliometric and other cumulative advantage processes, J.Amer.Soc.Inform.Sci. **27**, 292–306 (1976).

[2] M. Newman, *Networks: an introduction* (Oxford University Press, 2009).

[3] C. Cannings and J. Jordan, Random walk attachment graphs, Electronic Communications Probability **18** (2013).

[4] M. K. Hassan, L. Islam, and S. A. Haque, Degree distribution, rank-size distribution, and leadership persistence in mediation-driven attachment networks, Physica A: Statistical Mechanics and its Applications **469**, 23 (2017).

[5] W. H. Press, S. A. Teukolsky, W. T. Vetterling, and B. P. Flannery, Numerical recipes in C., Cambridge: Cambridge University (1992).

[6] J. R. Clough, J. Gollings, T. V. Loach, and T. S. Evans, Transitive reduction of citation networks, Journal of Complex Networks **3**, 189 (2015).

[7] V. Vasiliauskaite, and T. S. Evans, Making communities show respect for order, (2019) `arXiv:1908.11818`.
